# A general method for directly phasing diffraction data from high-solvent-content protein crystals

**DOI:** 10.1107/S2052252522006996

**Published:** 2022-08-13

**Authors:** Richard Lawrence Kingston, Rick P. Millane

**Affiliations:** a School of Biological Sciences, University of Auckland, 3a Symonds St, Auckland City, Auckland 1010, New Zealand; b Computational Imaging Group, Department of Electrical and Computer Engineering, University of Canterbury, Christchurch, New Zealand; University of Michigan, USA

**Keywords:** imaging, structure determination, phasing, protein crystals, high-solvent-content crystals, solvent flatness, X-ray crystallography

## Abstract

A procedure is described for direct phase determination in protein crystallography, applicable to crystals with high solvent content. The procedure requires only the diffraction data and an estimate of the solvent content as input. Direct phase determination is treated as a constraint satisfaction problem, in which an image is sought that is consistent with both the diffraction data and generic constraints on the density distribution in the crystal. The problem is solved using an iterative projection algorithm, the *Difference Map* algorithm, which has good global convergence properties, and can locate the correct solution without any initial phase information. Computational efficiency is improved by breaking the problem down into two stages; initial approximation of the molecular envelope at low resolution, followed by subsequent phase determination using all of the data. The molecular envelope is continually updated during the phase determination step. At both stages, the algorithm is initiated with many different and random phase sets, which are evolved subject to the constraints. A clustering procedure is used to identify consistent results across multiple runs, which are then averaged to generate consensus envelopes or phase sets. The emergence of highly consistent phase sets is diagnostic of success. The effectiveness of the procedure is demonstrated by application to 42 known structures of solvent fraction 0.60–0.85. The procedure works robustly at intermediate resolutions (1.9–3.5 Å) but is strongly dependent on crystal solvent content, only working routinely with solvent fractions greater than 0.70.

## Abstract 2

This paper describes a practical method for *ab initio* phasing of diffraction data collected from high-solvent-content protein crystals (solvent content >70%). Unlike traditional direct methods, this procedure leverages the powerful constraint of solvent flatness, and works at the modest resolutions (2.0–3.5 Å) and with the large numbers of atoms, that are typical of protein crystallography.

## 1. Introduction

Although crystallographic imaging of protein molecules has become relatively routine, the problem of phase determination can still present difficulties. For X-ray crystallography, phase determination based on isomorphous replacement and anomalous diffraction (Taylor, 2010 ▸; Hendrickson, 2014 ▸) is highly effective but can require substantial experimental effort. For the emergent technique of electron crystallography, experimental approaches for phase determination remain in development (Martynowycz *et al.*, 2020 ▸). Existing knowledge of protein structure can often be leveraged to overcome the phase problem, using the method of molecular replacement (Scapin, 2013 ▸). However, this approach suffers from the problem of model bias (Adams *et al.*, 1999 ▸; DiMaio *et al.*, 2011 ▸) and may not always succeed.

*Ab initio* phasing in protein crystallography – the formation of an image direct from the diffraction data, without requiring any ancillary experimental measurements or detailed knowledge of the target structure – is therefore a problem of both practical and theoretical importance. In chemical crystallography, where the molecules being characterized are much smaller than proteins, *ab initio* phasing is routinely achieved using computational ‘direct methods’ which are grounded in probability theory and exploit the truly atomistic character of the image (Giacovazzo, 1999 ▸). Where atomic-resolution diffraction data are available these methods can be used for modestly sized proteins (Usón & Sheldrick, 1999 ▸), and other novel methods of phase determination are enabled (see *e.g.* McCoy *et al.*, 2017 ▸; Coelho, 2021 ▸). Such methods are, however, not generally applicable. A large body of early work focused on *ab initio* determination of the molecular envelope at very low resolution. While this was achieved using a variety of approaches (Subbiah, 1991 ▸, 1993 ▸; David & Subbiah, 1994 ▸; Urzhumtsev *et al.*, 1996 ▸; Andersson & Hovmöller, 1996 ▸; Podjarny & Urzhumtsev, 1997 ▸; Müller *et al.*, 2006 ▸; Lunin *et al.*, 2007 ▸; Urzhumtsev *et al.*, 2008 ▸; Lunin *et al.*, 2012 ▸), this work was not extended to provide a complete solution of the phase problem.

While *ab initio* phase determination is uncommon in protein crystallography, procedures for improving and extending experimental phase estimates find near universal application. In these ‘density modification’ procedures (Podjarny *et al.*, 1996 ▸; Zhang *et al.*, 2012 ▸), an initial image, typically calculated with experimentally derived phase estimates, is iteratively modified, applying generic constraints on the density in real space, and enforcing the measured diffraction amplitudes in Fourier space. Among the constraints applied to the density, the most ubiquitous are solvent flatness (the solvent region should be effectively featureless) (Schevitz *et al.*, 1981 ▸; Wang, 1985 ▸), histogram equivalence (the protein region should have a characteristic density value distribution) (Lunin, 1988 ▸; Harrison, 1988 ▸; Yong *et al.*, 1990 ▸; Lunin & Skovoroda, 1991 ▸; Lunin & Vernoslova, 1991 ▸) and symmetry equivalence (if multiple copies of a molecule are present in the asymmetric unit, their associated densities should be the same) (Main & Rossmann, 1966 ▸; Crowther, 1967 ▸, 1969 ▸; Bricogne, 1974 ▸; Colman, 1974 ▸). The application of solvent and histogram constraints requires knowledge of the molecular envelope, while application of symmetry constraints also requires understanding the nature and position of any symmetry elements present. Having some initial phase estimates allows these issues to be resolved at the outset (Wang, 1985 ▸; Lawrence, 1991 ▸; Kleywegt & Read, 1997 ▸). While generally effective, iterative density modification (IDM) procedures have a small radius of convergence, in the sense that they converge to a good solution only if initiated with reasonably good phase estimates. With poor initial phase estimates, conventional algorithms may quickly reach a *fixed point* in which the algorithm stagnates, and the true density cannot be derived from the fixed point.

A fundamental question is whether the real-space image constraints used in IDM could provide enough information to overcome the absence of the phases in protein crystallography and render a unique solution to the phase problem. If this were the case, it should be possible to use these constraints for *ab initio* phase determination, if a better algorithm for locating the solution were devised. The problem of uniqueness has been discussed by a number of authors (Crowther, 1969 ▸; Bricogne, 1974 ▸; Baker *et al.*, 1993 ▸; Millane, 1993 ▸; Miao *et al.*, 2000 ▸; Elser & Millane, 2008 ▸; Millane & Lo, 2013 ▸; Millane & Arnal, 2015 ▸). Here we focus on the solvent flatness constraint. In the simplest description, since the Fourier amplitudes and phases are both necessary and sufficient to calculate the density, and can be considered mutually independent at non-atomic resolution, loss of the phases amounts to loss of half the information required to reconstruct the density. Therefore, at a minimum, a twofold redundancy in the density is needed for the density to be uniquely related to the Fourier amplitudes (*i.e.* the number of degrees of freedom of the density in the asymmetric unit must be halved). For example, if the molecular envelope were known, and the solvent volume fraction exceeds one half, then the degrees of freedom of the density function are halved by the solvent flatness constraint. In this case the protein density should be uniquely determined by the Fourier amplitudes alone. Of course, in the *ab initio* case, the envelope is unknown. However, Millane (Millane & Arnal, 2015 ▸) has shown that a unique solution is expected even if only the *volume* of the molecular envelope is known, and is greater than 50% of the unit-cell volume, although the reconstruction problem is clearly more difficult in this case. In practice, a phase retrieval algorithm would not be expected to work at this theoretical limit. One reason for this is deficiencies in the data. In particular, the ultra-low-resolution diffraction data, central to the definition of the molecular envelope, are prone to systematic artifacts, and are often left unmeasured. However, *ab initio* phase determination, based on the solvent flatness constraint, should be feasible for diffraction data from crystals with a solvent volume fraction somewhat greater that 50%. The challenge is to develop an algorithm that can routinely find the solution, which is the topic of this paper.

An approach to *ab initio* phasing similar to IDM should be effective, if the radius of convergence could be extended, so that convergence to the correct density is achieved when the algorithm is initiated with random phases (Millane, 1990 ▸). This idea has been pursued in the past decade, buoyed by the success of such approaches in optical and single-particle imaging (Miao & Sayre, 2000 ▸; Donatelli *et al.*, 2015 ▸, 2017 ▸; Ekeberg *et al.*, 2015 ▸; Grant, 2018 ▸). The basis of the approach is the use of iterative projection algorithms (IPAs). These are algorithms resembling conventional IDM, that iterate between real space and Fourier space, while incorporating constraints in each domain. However, the operations performed at each iteration are more complicated than those performed in conventional IDM. The key advantage of these algorithms is that they have good global, as opposed to local, convergence properties. This makes them excellent candidates for *ab initio* phasing in protein crystallography in cases where there is sufficient real-space information [*e.g.* high solvent content or non-crystallographic symmetry (NCS)] to define a unique solution with only the diffraction amplitude data.

The general approach was first posited by Millane (Millane, 1990 ▸) and its potential demonstrated by successful *ab initio* phasing from noisy, simulated diffraction amplitudes from an icosahedral virus crystal with fivefold NCS (Millane & Stroud, 1997 ▸; van der Plas & Millane, 2000 ▸), building on earlier work with virus crystals that used conventional IDM (Chapman *et al.*, 1998 ▸; Rossmann, 1995 ▸). A formal presentation of these kinds of algorithms as IPAs was given by Elser (Elser, 2003*a* ▸,*b* ▸), which put the approach on a more rigorous theor­etical basis. Elser also described a generally applicable IPA, termed the *Difference Map* (*DM*) algorithm (Elser, 2003*b* ▸), which we employ in this study (see Section 2.1, below). Another widely used IPA is the *Hybrid Input Output* (*HIO*) algorithm, first described by Fienup (Fienup, 1982 ▸). A review of IPAs, and their potential applications in protein crystallography, including their connections with IDM, was presented by Millane and Lo (Millane & Lo, 2013 ▸). The first applications to experimental diffraction data from protein crystals used solvent flatness constraints in conjunction with the *HIO* algorithm (Liu *et al.*, 2012 ▸) or solvent flatness and NCS constraints in conjunction with the *DM* algorithm (Lo *et al.*, 2015 ▸, 2016 ▸) to successfully recover the electron density for previously determined test cases. However, while these approaches began with random phases, they are not truly *ab initio*, as they assumed some low-resolution envelope information. A very significant advance was made by Su *et al.* who incorporated envelope determination and refinement into the *HIO* algorithm and showed successful recovery of the electron density for a number of protein crystals with solvent volume fractions around 0.70 or greater (He & Su, 2015 ▸; Jiang *et al.*, 2018 ▸; He & Su, 2018 ▸; Jiang *et al.*, 2019 ▸). These studies represent the current state-of-the-art using this approach, but it has been unclear how generally this procedure could be made to work, and the dependencies on resolution or other factors.

In this paper, we present a practical method to routinely phase diffraction data of modest resolution (1.9–3.5 Å) from protein crystals with high solvent content (solvent volume fraction greater than ∼0.70). The procedure works in an unsupervised fashion, requiring only the diffraction data, and an estimate of the solvent content as input. The method successfully addresses the difficult problem of envelope determination and incorporates several differences from previous approaches. Firstly, we employ the *DM* algorithm, which can accommodate restraints more flexibly than the *HIO* algorithm. Secondly, we employ a computationally efficient two-stage procedure, in which an approximate molecular envelope is determined from calculations at low resolution, facilitating subsequent phase determination using all available data. Finally, for both envelope and phase determination, the algorithm is initiated multiple times with random phase sets, and clustering procedures are used to identify and combine consistent results from multiple runs. This is a key aspect of the procedure, as conventional metrics may not indicate that a solution has been found, whereas clustering reliably identifies solutions. The emergence of highly consistent phase sets during the second step of the procedure effectively confirms the solution has been located. The method was tested through application to a total of 42 diffraction data sets from the Protein Data Bank (PDB), from crystals in a variety of space groups, and having solvent fractions 0.60–0.85. As currently parameterized, the algorithm successful recovered the electron density for 87% of the data sets with solvent fraction >0.75 (13/15 cases) and 73% of the data sets with solvent fraction >0.70 (22/30 cases). Our study establishes the approach as a viable phase determination technique, and the creates a critical benchmark, against which future development of the algorithm can be measured. Code implementing the procedure has been made publicly available on GitHub (https://github.com/rlkingston/IPA).

## 2. Methods

### 2.1. Iterative projection algorithms

Given the measured Fourier amplitudes and sufficient constraints on the density, the solution to the phase problem will be unique. However, a phase retrieval algorithm may fail to find the solution because the associated optimization problem is highly non-convex and location of the correct density is nontrivial. Our approach is to use iterative projection algorithms with good global convergence properties to find a unique solution, if it exists. Here, we briefly review IPAs and the *DM* algorithm that we use. The reader is referred to (Millane & Lo, 2013 ▸) for more information.

It is convenient to represent the electron density as a point in an *N*-dimensional Euclidean vector space, where the elements of a vector **x** are the values of the density at the *N* grid points in the unit cell (or the asymmetric unit). The reconstruction problem is cast as a constraint satisfaction problem. In the case at hand, we have constraints in both Fourier space (that the calculated and measured Fourier amplitudes should agree) and in real space (that the density should conform to the expected distributions in the protein and solvent regions). These are the only real-space constraints used in this study, though obviously others might be employed. If the solution to the problem is unique, then a density that satisfies all the constraints (or lies in the intersection of the corresponding constraint sets in the vector space) is the correct density. The problem then is to find a point in the intersection of the constraint sets. This is referred to as a constraint satisfaction problem. Solving the problem is difficult because one of the constraints, the Fourier magnitude constraint, is non-convex (Elser, 2003*a* ▸; Millane & Lo, 2013 ▸). Iterative projection algorithms have been shown to be effective in solving non-convex constraint satisfaction problems (Elser *et al.*, 2007 ▸).

The constraints for our problem are collected into two sets, one in real space, denoted *A*, and one in Fourier space, denoted *B*. IPAs make use of *projections*. The projection of a density **x** onto a constraint set *C*, denoted *P_C_* **x**, is the density that is closest (in the Euclidean, or least-squares, sense) to **x** and satisfies the constraint *C*. The projection then corresponds to making the smallest change to the (current) density such that is satisfies the constraint.

Once initiated, in our case with a random density, an IPA generates a sequence of densities, with the objective of converging to the true density. The sequence is defined by an update rule that takes the density at iteration *n*, **x**_*n*_, to that at iteration *n* + 1, **x**_*n* + 1_, and the update rule consists of a combination of projections of ***x***_n_.

It can be seen immediately that the iterative density-modification procedure commonly used in protein crystallography is an IPA with update rule

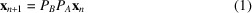


*i.e.* the density is adjusted to exactly satisfy the real-space constraints, then to exactly satisfy the Fourier space constraints, and the cycle repeated. This algorithm is often termed the *Error Reduction* (*ER*) algorithm (Fienup, 1982 ▸). The *ER* algorithm, however, does not have good global convergence in the presence of non-convex constraints, and will quickly stagnate at a density that does not satisfy both constraints. This is the reason that conventional phase improvement algorithms are successful only if started with good initial phase estimates and are not useful for *ab initio* phasing.

There are various other more sophisticated IPAs that have better global convergence properties in the presence of non-convex constraints (Marchesini, 2007 ▸; Millane & Lo, 2013 ▸). One of these is the *Difference Map* (*DM*) algorithm (Elser, 2003*a* ▸), which we employ in this paper. The update rule for the *DM* algorithm is 

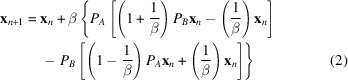

where β is an adjustable parameter with −1 < β < 1. The parameter β controls the ‘relaxation’ of the projections and affects the behavior of the algorithm in terms of both speed of convergence and the degree to which it searches the parameter space. Its value, however, cannot be simply related to algorithm performance. Suitable values for β are problem dependent, and we detail the values used in our application below. Note that changing the sign of β effectively reverses the order in which the projections *P_A_* and *P_B_* are applied in (2).

At each iteration, the *DM* algorithm generates two solution estimates 

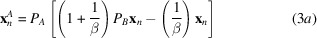




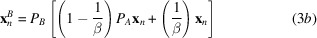

which satisfy the constraints *A* and *B*, respectively, but not necessarily both. If the two solution estimates are equal, then the algorithm has converged, and either is a solution to the constraint satisfaction problem. These two solution estimates are combined via (2), to generate the updated density, **x**_*n* + 1_, which is not itself an estimate of the solution.

At any iteration, the root-mean-square deviation between the two solution estimates (δ_*DM*_) can be evaluated, which provides a simple measure of algorithm convergence.

### 2.2. Apodization of the Fourier amplitude data

As in our previous work (Lo *et al.*, 2015 ▸), the effective resolution of the reconstruction is controlled by apodizing the Fourier amplitude data with a Gaussian function. The weighting function applied is

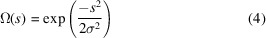

where *s* is the distance from the origin in Fourier space (the magnitude of the scattering vector = 1/resolution, Å^−1^), and σ is the standard deviation of the Gaussian function (Å^−1^). For convenience we often express the width of the Gaussian function in terms of the half width at 1/100th of maximum height = 3.03σ (Å^−1^), or the corresponding resolution.

The use of a Gaussian apodization function avoids introducing ringing artifacts into the Fourier synthesis that would accompany the use of a simple step function to limit resolution. During phase determination we find that gradually increasing the value of σ, and hence the effective resolution of the reconstruction, aids convergence to the solution.

### 2.3. Fourier space constraints, projections and agreement metrics

In Fourier space, the constraints are the measured Fourier amplitudes. The corresponding projection onto the constraint is simple and consists of Fourier transforming the current density; replacing the Fourier amplitudes with the measured Fourier amplitudes (after applying the apodization function, and scaling, as appropriate); and transforming back to real space. We also need to consider treatment of the Fourier amplitudes for which data are not available (the unmeasured or ‘missing’ diffraction data) – termed the *unconstrained modes* in the image processing field. If these amplitudes are allowed to evolve without constraint, excessively large values may result which can produce severe distortions in the reconstructed density. To prevent the evolution of physically unrealistic Fourier amplitudes, checks are performed on the amplitudes of the missing data during each projection operation, based on Wilson intensity statistics (Rogers, 1965 ▸). If a reconstructed Fourier amplitude is improbably large (probability <5 × 10^−6^), it is reset near the expected value at the relevant resolution.

At each iteration, the agreement between the relevant solution estimate (3*a* [Disp-formula fd3a]) and the Fourier space constraints is evaluated in the usual way, by computing a correlation coefficient, or *R* factor, between measured and reconstructed Fourier amplitudes.

### 2.4. Real-space constraints, projections and agreement metrics

In real space, the constraints utilized are the expected density distributions in the protein and solvent regions. The protein region has a generic density distribution which can be reliably predicted, while the solvent region is essentially featureless, and should have a one–point distribution. The corresponding projection onto the constraint set consists of making the minimum change to the density required to transform the distributions in both regions into the expected distributions. Hence, the constraints are those routinely applied in conventional crystallographic phase refinement – ‘solvent flattening’ in the solvent region (Schevitz *et al.*, 1981 ▸; Wang, 1985 ▸), and ‘histogram matching’ in the protein region (Lunin, 1988 ▸; Harrison, 1988 ▸; Yong *et al.*, 1990 ▸; Lunin & Skovoroda, 1991 ▸; Lunin & Vernoslova, 1991 ▸). However, while the projections are the same, the way they are incorporated into the phase retrieval algorithm is fundamentally different [compare equations (1) and (2)].

Application of these constraints requires definition of the molecular envelope, *i.e.* a binary partitioning of the image into protein and solvent regions, consistent with the overall solvent fraction. Since the molecular envelope is *a priori* unknown, it must be determined as part of the reconstruction procedure. Simple thresholding algorithms based on local averaging of the density function (Wang, 1985 ▸; Leslie, 1987 ▸) are frequently used to define the molecular envelope in protein crystallography. For direct phase determination, we find that determining the molecular envelope based on the local variance of the density (Abrahams & Leslie, 1996 ▸), rather than the local mean density, gives uniformly better results, and we have adopted this procedure throughout. To define the envelope, the variance map is filtered using a triweight function of defined radius (*r*_0_), 

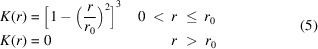

where *r* is the distance to the current grid point. The filtered map is thresholded to generate an envelope with the desired volume fraction. The envelope is updated at each iteration of the algorithm, based on one of the solution estimates (3*b* [Disp-formula fd3b]).

With the envelope defined, the projection is carried out by setting the density in the solvent region equal to its mean value. The density in the protein region is transformed to achieve the desired distribution (Harrison, 1988 ▸; Lunin & Vernoslova, 1991 ▸), shifting the overall mean to its expected value, while preserving the variance.

The ‘histogram matching’ operation carried out in the protein region requires that the expected density distribution is known. While it is well established that electron density distributions for proteins are generic they are strongly influenced by both data resolution, and the overall isotropic displacement parameter (*B* factor) of the data (Yong *et al.*, 1990 ▸; Lunin & Skovoroda, 1991 ▸). Our approach is to select a suitably matched reference structure from the PDB, in a case-dependent fashion, for calculation of the expected distribution. This requires robust estimation of the overall *B* factor of the target structure, which is *a priori* unknown. The overall isotropic *B* factor is estimated from the diffraction data using an approach based on the Patterson origin function (Blessing & Langs, 1988 ▸). Note that the experimental data are sometimes apodized to control the effective resolution of the image. In this case the data used to calculate the reference distribution are apodized in an equivalent fashion.

At each iteration, the agreement between the relevant solution estimate (3*b* [Disp-formula fd3b]) and the real-space constraints is evaluated. In the solvent region, the variance of the reconstructed density is used as a measure of solvent flatness. In the protein region, the Wasserstein distance (‘Earth-Movers distance’) between the reconstructed and target density value distributions is used as a measure of histogram agreement. The first Wasserstein distance, denoted *W*_1_(*P*,*Q*) for two random variables *P* and *Q*, is a true distance metric for probability distributions (Panaretos & Zemel, 2019 ▸), and is conveniently evaluated from the cumulative distribution functions of the two distributions, *F_P_* and *F_Q_* as

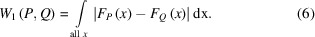




### 2.5. Metrics for evaluating algorithm performance on test cases

Testing and evaluation of the envelope and phase determination procedures requires suitable metrics for computing agreement with the known envelope or phase set.

To compare two binary-valued molecular envelopes, the correlation coefficient is used, which can be calculated as (Warrens, 2008 ▸), 

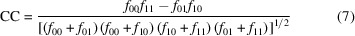

where *f*_00_ and *f*_11_ are the proportions of grid points that match in the solvent and protein regions, respectively, and *f*_01_ and *f*_10_ are the proportions that do not match. The correlation coefficient (for binary-valued functions often termed the Matthews correlation coefficient, or the Pearson or Yule Phi coefficient) has a value of 1 if the envelopes match exactly and a value of 0 if the two envelopes are statistically independent.

For measuring agreement between two phase sets, we employ the weighted mean absolute phase difference, where the weights are derived from any apodization scheme (4) being applied to the Fourier amplitudes. Fourier coefficients calculated from refined atomic models incorporated no correction for the scattering of the bulk solvent.

### 2.6. Origin ambiguity and inversion ambiguity

For direct phase determination, initiated with a random phase set, the crystallographic origin is effectively selected arbitrarily, and both binary envelopes and density functions (or the correspondent phases in Fourier space) need to be referenced to a common origin before comparative statistics are calculated, or any form of averaging performed (Lunin *et al.*, 2012 ▸). The alternate origin choices have been tabulated for all space groups (see *e.g.* Giacovazzo, 1999 ▸) and FFT-based image registration [the ‘phased translation function’ (Read & Schierbeek, 1988 ▸)] is used to identify the permitted origin shift that results in the best agreement in both cases.

A related problem is that the image constraints being applied are equally well satisfied by the true density, or the map obtained by inversion of the true density. The same issue manifests in many experimental phase determination procedures [discussed in Matthews (2007 ▸); Wang *et al.* (2007 ▸); McCoy & Read (2010 ▸)]. Hence, any solution generated may have the incorrect hand. In any one of the 22 chiral space groups, inversion of the density function is associated with a change in space group (and hence generation of a density map with incorrect hand is diagnostic of an incorrect space group choice, see below). In the remaining 43 achiral space groups relevant to protein crystallography (Nespolo *et al.*, 2018 ▸), either the solution or its inverse may be generated, and both possibilities must be checked when comparing either binary-valued envelopes or real-valued density functions. In an unknown case, potential solutions to the phase determination problem would need to be visually inspected to determine the correct hand This step may be amenable to automation (Condado *et al.*, 2022 ▸).

### 2.7. Procedures for algorithm optimization

To test and optimize our methods, we selected 42 test cases for study from existing PDB depositions. The selection was made at the outset of the investigation before the phasing procedure was fully developed. Data sets were selected to provide a range of relevant solvent contents (60–85%), and a variety of space groups, but otherwise the selection was essentially random. Relevant details of the test cases are given in Supplementary Table S1.

Our aim was to develop a common phasing procedure that could be applied to all test cases without user intervention. To do this we optimized various parameters associated with the envelope and phase determination steps on a small subset of the test cases. Some of the more critical parameters investigated were the values of β used in the *DM* algorithm; the low-resolution cutoff applied to the diffraction data; the nature of the apodization scheme applied to the diffraction data; the filter radius used for determination of the molecular envelope; and the total number of iterations. Because of the complex and multivariate nature of the problem, we made extensive use of factorial experiments and orthogonal-array-based experimental designs (Hedayat *et al.*, 1999 ▸) to identify the most productive regions of the parameter space. These experiments consisted of running envelope or phase determination repeatedly, using the same set of pseudo-random starting points, while varying the parameters according to the experimental design.

In general terms we found that schemes in which β is systematically alternated between two values are more effective than schemes in which β is held fixed throughout; that omission of the ultra low-resolution data aids convergence to the solution, as previously noted (Jiang *et al.*, 2018 ▸); and that gradual shrinking of the filter radius (5) during envelope determination aided coalescence of the molecular envelope. In addition, we found that once a solution is located, executing the *DM* algorithm with a negative β value (which amounts to reversing the order in which the constraints are applied), generally results in some additional phase improvement.

The experiments resulted in development of a common protocol (described below) that was subsequently applied to all test cases without variation, generating the results described in the paper.

### 2.8. Parameterization of the algorithm for envelope determination

For envelope determination the *DM* algorithm, initiated with a fully random phase set, is run 50 times for each test case. Each of these 50 runs consists of 1475 iterations followed by 25 iterations of the ER algorithm, for a total of 1500 iterations. The parameter β of the *DM* algorithm is assigned one of two values (0.72 or 0.78) alternating between them at each iteration. The Fourier amplitude data are heavily apodized with a Gaussian function that is unchanged throughout (σ = 0.091 Å^−1^, hence reaching 1/100th of maximum height at 3.6 Å resolution). A low-resolution cutoff of 25 Å resolution is applied to the dataset, with the lower resolution terms being treated as missing throughout. The molecular envelope is updated at each iteration, with the radius of the triweight filter function (5) shrinking from 10.8 to 8.0 Å across the first 1000 iterations of the *DM* algorithm, and a constant 8.0 Å thereafter.

Conservatively, we consider 3.6 Å to be the effective resolution limit of the data, and grid the density maps at 2/5 of this limit, allowing for relatively rapid evaluation of the Fast Fourier Transform during envelope determination.

### 2.9. Parameterization of the algorithm for phase determination

For phase determination the *DM* algorithm, initiated with a fully random phase set and a consensus molecular envelope (see below), is run 20 times for each test case. The molecular envelope is held fixed for the first 10 iterations and subsequently updated at each iteration, using a fixed radius of 8.0 Å for the triweight filter function (5). A low-resolution cutoff of 25 Å is again applied to the diffraction data, with the lower resolution terms being treated as missing.

Over 7200 iterations of the DM algorithm, the effective resolution of the reconstruction is gradually increased by apodizing the diffraction amplitude data with a steadily broadening Gaussian function (4). This occurs over a total of 30 steps (240 iterations of the DM algorithm at each step). At the first step, the apodization function has σ = 0.16 Å^−1^ (hence reaching 1/100th of maximum height at 2.1 Å resolution). At each subsequent step, the area under the Gaussian function (evaluated up to the full resolution of the dataset) is increased by a fixed increment, so that by the final step, the data are essentially unmodified (Supplementary Fig. S1). Throughout, the parameter β is assigned one of two values (0.675 or 0.800), alternating between them every 60 iterations.

After the first 7200 iterations, the *DM* algorithm is run for a further 200 iterations using the diffraction data with no apodization applied. The first 100 of these iterations are performed with β = 0.75 and the second 100 with β = −0.55. Then 25 iterations of the *ER* algorithm are performed. This sequence of 225 iterations is repeated a total of four times, and the run then concludes.

Hence overall, each run extends for 8100 iterations, the first 7200 with steady removal of the apodization and involving only the *DM* algorithm, and the last 900 with no apodization, interspersing the *DM* and *ER* algorithms.

### 2.10. Clustering algorithms, and the computation of consensus envelopes and phase sets

Clustering of both molecular envelopes and phase sets is used to identify similar outputs resulting from multiple runs of the algorithm. From clusters of similar solutions, a ‘consensus’ envelope or phase set is produced by averaging all members of the cluster. Details of the procedure differ for binary-valued envelopes and phases sets, but we begin with some general comments

The pairwise distances (*d*) between all inputs are first calculated, providing the basis for the clustering procedure. With *N* inputs, there are a total of *N*(*N* − 1)/2 unique distances. For binary envelopes we take *d* = (1 − CC^2^)^1/2^, where CC is the map correlation coefficient (7). For phase sets, we take *d* = the mean absolute phase difference. At the same time, the translations required to put each pairing on a common origin are also evaluated. The standard data clustering algorithm *DB-SCAN* (Schubert *et al.*, 2017 ▸) is then used to cluster the inputs. *DB-SCAN* has two parameters, the minimum number of points (*minPoints*) required to form a dense region, and the threshold distance (ɛ) for the clustering procedure.

#### 2.10.1. Envelopes

For clustering of the molecular envelopes, *minPoints* is set to 5 (*i.e.* 10% of the input set size, which was the 50 envelopes) while the threshold distance, ɛ, is set to the 4th percentile of the distribution of pairwise distances. This is because the values of the correlation coefficient vary widely across the tested diffraction data sets, and there is no absolute threshold which will work for all cases.

Once the clustering has been performed, the translational shifts needed to bring the envelopes onto a common origin are applied, and the consensus envelope is calculated for each cluster by taking the modal value for each sample point in the envelope, across all members of the cluster. The consensus envelope is subsequently edited using a previously published connectivity algorithm (Hunt *et al.*, 1997 ▸), erasing small islands, and filling small voids, that can sometimes be created by the averaging procedure.

#### 2.10.2. Phases

For clustering of phase sets, *minPoints* is set equal to 2, while the threshold distance, ɛ, is set to 40°–50° mean absolute phase difference. These settings discriminate correct from incorrect solutions for all test cases.

A consensus phase set is obtained by calculating the mean phase across all members of the cluster, applying any needed origin shifts. The consistency of the phases sets within a cluster is evaluated using the sample circular variance (Fisher, 1993 ▸).

### 2.11. Practical implementation

The procedures described here have been implemented in a program called *IPA* (*Iterative Projection Algorithms for protein crystallography*) using the Clipper C++ crystallographic library (Cowtan, 2003 ▸) for all the core crystallographic tasks. The program is written in C++ with some ancillary routines in modern Fortran. Our implementation automates the procedures described in this paper, and requires only the measured diffraction data and the solvent content as compulsory input. The procedure has been parallelized (see *Discussion* [Sec sec4]) which can greatly reduce time to completion when multiple CPUs and cores are available for the calculation. The behavior of the program *IPA* is controlled by a single experimental parameter file. Use of the default parameter file will reproduce the protocol described in the paper exactly, however the user may elect to vary the protocol by editing the parameter file. The code is available on GitHub (*IPA* version 1.0.0, https://github.com/rlkingston/IPA). The details of the implementation will be described in a subsequent publication.

## 3. Results

### 3.1. Preliminaries

The 42 test cases employed in the study are detailed in Supplementary Table S1, arranged in order of decreasing solvent fraction (0.85–0.60). Our intent was to establish a general procedure for directly phasing diffraction data from high-solvent content protein crystals. As described above, initial development work suggested that a two-stage procedure would be the most computationally efficient, in which a molecular envelope was approximated using calculations at low resolution, and subsequently used to initiate phase determination at higher resolution. Following this initial development work, we applied a common protocol to all test cases to benchmark the procedure.

The *DM* algorithm is used both to coalesce an envelope in the first stage, and to locate a complete solution to the phase problem in the second stage. During both stages, the constraints on the density are those used in conventional density-modification procedures, although the way that the constraint satisfaction problem is attacked is dramatically different (see *Methods* [Sec sec2]). In the solvent region the density should be featureless (*i.e.* have a one-point distribution), while in the remainder of the image the density distribution should be characteristic of proteins. The molecular envelope, required for application of these real-space constraints is continually updated, based on one of the solution estimates at each iteration of the algorithm.

The solvent fraction assumed for each test case is calculated from the molecular structure. In an unknown case, this would need to be estimated through analysis of the crystal packing density (Weichenberger *et al.*, 2015 ▸). Besides the diffraction data, the solvent fraction is the only other information required as input to the phase determination procedure.

To establish the expected protein density distribution for each test case, the overall isotropic *B* factor is estimated from the experimental diffraction amplitudes, using an approach based on the Patterson origin function (Blessing & Langs, 1988 ▸). This procedure returns values consistent with refined atomic models (Supplementary Table S1). Based on the estimated *B* factor and the resolution limit of the data set, a reference structure is automatically selected for calculation of the expected electron-density distribution (histogram). In practice, electron-density histograms calculated from one of 10 reference structures were used for all the calculations described here (Supplementary Table S1).

### 3.2. Envelope determination

#### 3.2.1. Envelope generation

To generate a set of molecular envelopes, the *DM* algorithm, initiated with a fully random phase set, was executed 50 times for each test case. The diffraction amplitude data are heavily apodized to strongly downweight the high-resolution data (all datasets were multiplied with a Gaussian function with σ = 0.091 Å^−1^, hence reaching 1/100th of maximum height at 3.6 Å resolution). Each run extends for 1475 iterations of the *DM* algorithm, followed by 25 iterations of the *ER* algorithm, with the apodization unchanged throughout.

**Figure 1:**
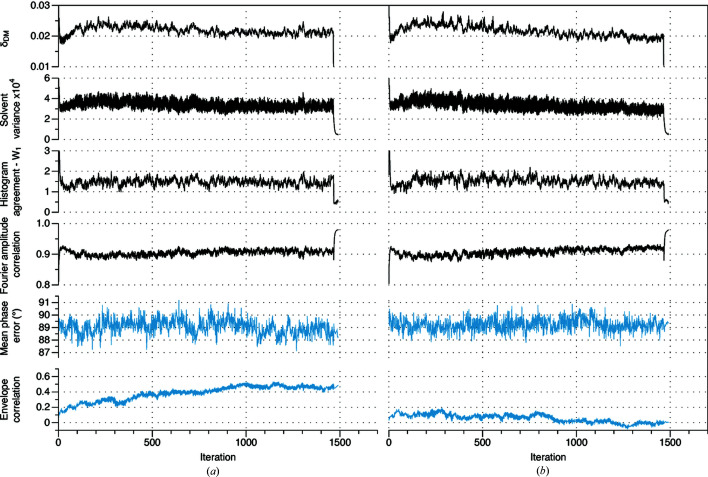
Molecular envelope generation for PDB entry 5b2c (Kubota *et al.*, 2016 ▸) (resolution 2.2 Å, solvent fraction 0.726). (*a*) The trajectory of a successful run. (*b*) The trajectory of an unsuccessful run. The two runs were initiated with different random phase sets. Plotted from top to bottom, as a function of iteration, are the convergence indicator of the *DM* algorithm (δ_DM_); the variance in the solvent region; the Wasserstein distance between reconstructed and reference histograms in the protein region; the correlation between reconstructed and measured Fourier amplitudes; the weighted mean absolute difference between reconstructed and model phases; and the correlation between the reconstructed and model envelopes. The metrics that could be followed during determination of an unknown structure are shown in black, while the metrics that assess agreement with the known solution are shown in blue.

Some typical trajectories for the *DM* algorithm during envelope generation are shown in Fig. 1 ▸ [test case 5b2c (Kubota *et al.*, 2016 ▸), resolution 2.2 Å, solvent fraction 0.73]. Displayed as a function of iteration are the measure of algorithm convergence (δ_*DM*_); the agreement measures with the real-space constraints (the solvent variance, and the Wasserstein distance between image and reference density value histograms), and the agreement measure with the Fourier space constraints (the correlation between measured and reconstructed Fourier amplitudes). These are the metrics that could be routinely followed during determination of an unknown structure. In addition, since refined atomic models are available for all test cases, we also follow the agreement between the reconstructed and model phases (evaluated using the weighted mean absolute phase difference) and the agreement between the reconstructed and model envelope (evaluated using the correlation coefficient). These latter metrics allow us to track algorithm performance across the runs.

These metrics vary in an erratic and apparently random fashion as the *DM* algorithm aggressively searches the parameter space for the vicinity of the solution. Irrespective of whether the system is in the vicinity of the solution or not, application of the *ER* algorithm at the end of each run gives rapid convergence to the nearest local minimum. These behaviors are quite typical of the *DM* and *ER* algorithms. However, the results shown in Fig. 1 ▸ also illustrate three more specific points, which are key to understanding the approach that has been adopted.

Firstly, a substantially correct molecular envelope frequently coalesces before a full solution to the phase problem is located. This is shown by the trajectory in Fig. 1(*a*) ▸. The agreement between the reconstructed and model envelope advances to a correlation coefficient of 0.57, which indicates the envelope is substantially correct. At the same time the weighted mean absolute difference from the model phases remains close to 90°, indicating that a solution to the phase retrieval problem has not yet been located.

Secondly, conventional metrics that can be calculated when determining an unknown structure, fail to robustly indicate that the envelope is evolving toward the correct answer. In the successful runs for test case 5b2c [*e.g.* Fig. 1(*a*) ▸] neither the agreement with the Fourier space constraints (the correlation with Fourier amplitudes), nor the agreement with the real-space constraints (the variance of the density values in the solvent region, and the Wasserstein distance between the observed and reference density value histograms in the protein region) indicate any progression toward a solution, even though a substantially correct envelope has formed by the end of each run.

Finally, the algorithm may or may not progress to a good envelope, dependent on the point at which it is randomly initiated. For the unsuccessful run shown in Fig. 1(*b*) ▸, a correct envelope does not coalesce over the course of the run, with a final correlation close to 0, which means the envelope is effectively unrelated to the true envelope.

Across all tested data sets, there is considerable variation in both the accuracy of the final envelopes, and the frequency with which accurate envelopes form, given a random starting point. Notwithstanding, in all cases the procedure generates some reasonable approximations to the molecular envelope. Some summary statistics (median and maximum agreement with the model envelope) are reported in Supplementary Table S2. In the case of novel structure determination, the challenge is to identify the accurate envelopes generated by the procedure, from the many inaccurate envelopes that are also generated.

**Figure 2:**
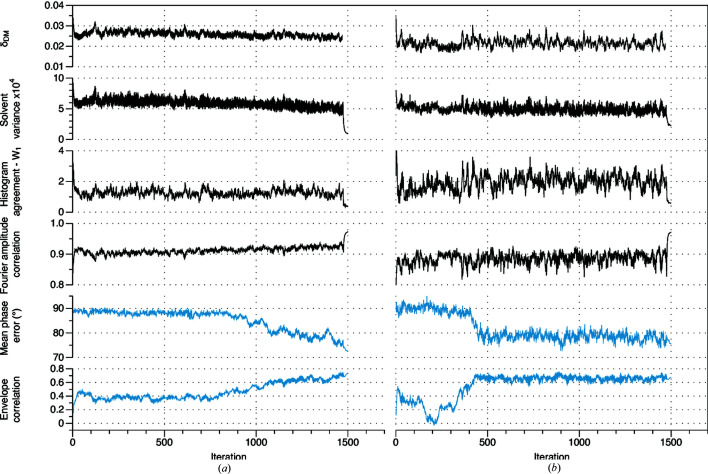
Molecular envelope generation and low–resolution phase determination. (*a*) The trajectory of a successful run for test case 3als (Hatakeyama *et al.*, 2011 ▸) (resolution 3.0 Å, solvent fraction 0.79). (*b*) The trajectory of a successful run for test case 4bex (Klejnot *et al.*, 2013 ▸) (resolution 2.8 Å, solvent fraction 0.73). Not only does a substantially correct molecular envelope coalesce over the course of each run but a satisfactory solution to the phase problem is located in both cases, with the weighted mean absolute difference with the model phases reducing to ∼75°. Plotted from top to bottom, as a function of iteration, are the convergence indicator of the *DM* algorithm (δ_DM_); the variance in the solvent region; the Wasserstein distance between reconstructed and reference histograms in the protein region; the correlation between reconstructed and measured Fourier amplitudes; the weighted mean absolute difference between reconstructed and model phases; and the correlation between the reconstructed and model envelope. The metrics that could be followed during determination of an unknown structure are shown in black, while the metrics that assess agreement with the known solution are shown in blue.

In favorable cases the procedure generates some very accurate envelopes (correlation coefficient with the model envelope of 0.75–0.87, Supplementary Table S2). In some of these instances (2rha, 3als, 4asn, 4bex, 4fzn), not only does the envelope coalesce, but a good solution to the phase problem is located at very low-resolution (some run trajectories for 3als and 4bex are displayed in Fig. 2 ▸). This is indicated by the clear progression away from random phase agreement. Interestingly, even in these favorable circumstances, the metrics that can be followed in an unknown structure determination give no indication that a solution has been located. The emergence of a correct low resolution phase set, as shown in Fig. 2 ▸ is quite uncommon, observed to occur for only 5 of the 42 test cases. Generally, despite the formation of a reasonable approximation to the envelope, a complete solution to the phase problem is not located at very low-resolution (see *e.g.* Fig. 1 ▸), even if the calculations are extended for many iterations. It is for this reason the first stage of our procedure is focused on the problem of binary envelope determination.

#### 3.2.2. Envelope clustering and averaging

The failure of conventional metrics to indicate the progression of the molecular envelope toward the correct result, coupled with the stochastic nature of that progression from a random starting point (Fig. 1 ▸, Supplementary Table S2), means that some novel method is required for identifying good approximations to the envelope. We solve this problem by introducing a clustering procedure to identify, and subsequently combine, the consistent results from the envelope determination runs. The underpinning principle is that good approximations to the envelope will agree with each other. In contrast, there are a multiplicity of incorrect envelopes capable of satisfying the volume fraction constraint, which should generally agree poorly with each other.

Envelopes are clustered based on the distance measure *d* = (1 − CC^2^)^1/2^, where CC is the correlation coefficient between a pair of envelopes (7), using the algorithm *DB-SCAN* (Schubert *et al.*, 2017 ▸). A consensus envelope is then generated for each cluster (see *Methods* [Sec sec2]). The results of clustering and averaging for all test cases are presented in Supplementary Table S2. These data show that the procedure effectively groups the best approximations to the envelope based on the pairwise distances. In all cases there is at least one cluster for which the accuracy of the consensus envelope closely approaches or exceeds the maximum accuracy of the individual envelopes. In many cases (21/42) that cluster is either unique (*i.e.* there is only one cluster), or unique with allowance for inversion (*i.e.* there are two clusters, related by inversion symmetry). An example is test case 3lii (Dvir *et al.*, 2010 ▸). For the 50 input envelopes the maximum correlation with the model envelope is 0.39. A single cluster of 16 envelopes is generated by *DB-SCAN*, with the consensus envelope having a correlation of 0.56 with the model envelope.

**Figure 3:**
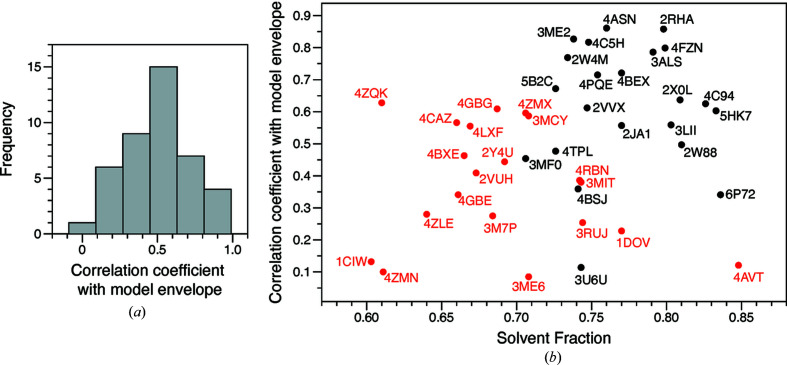
Overall results of envelope clustering. (*a*) The distribution of the correlation coefficient between the best consensus molecular envelope and the model envelope, for all 42 test cases (allowing for envelope inversion). (*b*) Scatter plot showing the correlation coefficient between the best consensus envelope and the model envelope as a function of crystal solvent content. PDB identifiers of the 42 test cases are indicated. Points colored black are the 22 data sets for which phases were subsequently successfully retrieved, points colored red are the 20 data sets for which phase retrieval was unsuccessful.

**Figure 4:**
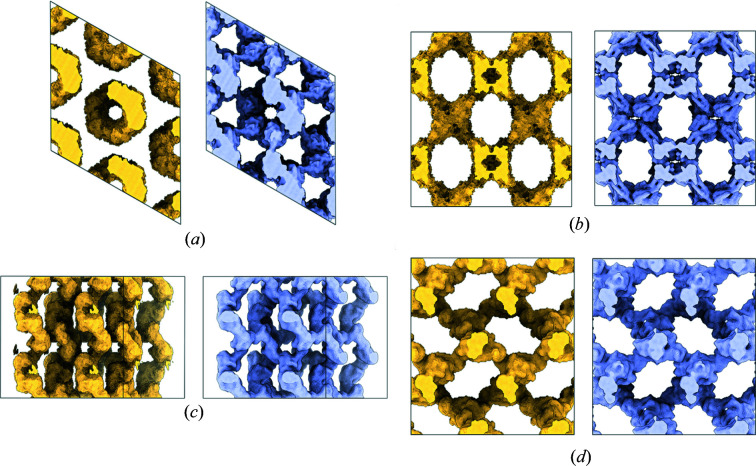
Comparison of consensus molecular envelopes with model envelopes. (*a*) Test case 5b2c (Kubota *et al.*, 2016 ▸) (solvent fraction 0.73, space group P6_1_), CC = 0.67, simple match between envelopes 87%. (*b*) Test case 5hk7 (Arrigoni *et al.*, 2016 ▸) (solvent fraction 0.83, space group *I*222), CC = 0.60, simple match between envelopes 90%. (*c*) Test case 4bex (Klejnot *et al.*, 2013 ▸) (solvent fraction 0.73, space group *P*3_2_21), CC = 0.77, simple match between envelopes 92%. (*d*) Test case 2rha (Joint Center for Structural Genomics, unpublished work) (solvent fraction 0.80, space group *P*4_3_2_1_2), CC = 0.86, simple match between envelopes 95%. Displayed in surface representation is the volume associated with 4 adjacent unit cells, from a single viewpoint. The consensus envelopes generated by the clustering and averaging procedure are shown in gold, and the model envelopes are shown in light blue. Figs. 4 ▸, 5 ▸ and 6 ▸ were prepared with the aid of UCSF *ChimeraX* (Pettersen *et al.*, 2021 ▸).

In other cases, one or two additional clusters are also generated. These can represent minor variations of the known envelope (*e.g.* 2vuh). Occasionally, they also represent envelopes with near–random correlation with the known envelope (*e.g.* 4gbe). In the latter case, the algorithm is repeatedly generating similar but incorrect envelopes. Nonetheless, clustering is still effective, as from 50 indiscriminable input envelopes, at most three consensus envelopes result, at least one of which is a fair to excellent approximation to the true envelope [Fig. 3 ▸(*a*)]. Some consensus envelopes generated by the procedure are compared to the model envelopes in Fig. 4 ▸. Interestingly, the accuracy of the final envelope is only weakly related to the solvent content [Fig. 3 ▸(*b*)].

Since our objective was to establish the feasibility of direct phase determination, we used only the best of the consensus envelopes [Supplementary Table S2, highlighted in light gray; Figs. 3 ▸(*a*) and 4 ▸] to initiate the phase determination step. For a real problem, if more than one envelope were generated (making allowance for inversion), then the phase determination step could be initiated using each candidate envelope in turn, and the solution would emerge with the correct envelope, as described below. Furthermore, there are several criteria that suggest which of the candidate envelopes is the best approximation, which could be used to put the envelopes in rank order. Among these criteria are the conformity with the desired volume fraction (due to the averaging process the consensus envelope may not have exactly the desired volume fraction) and the connectivity of the protein region (a good molecular envelope will be completely or nearly completely connected and should not contain disconnected ‘islands’). Inspection of the results in Supplementary Table S2 shows that many of the poorer envelopes are readily discriminated on this basis.

In summary, even though good approximations to the molecular envelope are routinely generated by the algorithm for almost all test cases, this is never indicated by conventional metrics, and many poor approximations to the envelope are also generated (Figs. 1 ▸ and 2 ▸). This problem is overcome by using clustering procedures to identify the consistent results from multiple runs, exploiting the uniqueness of the solution. Consistent envelopes are combined to generate a consensus envelope (Figs. 3 ▸ and 4 ▸), which is then used to aid phase determination.

### 3.3. Phase determination

Once the consensus envelope is generated, it is employed in the second stage of the procedure, to initiate phase determination at high resolution. The phase determination step employs the same basic *DM* algorithm as the envelope determination step but is parameterized quite differently (see *Methods*). The phase determination step involves more iterations (7200) than the envelope determination step, during which the resolution of the reconstruction is gradually increased, via the apodization function that is applied to the Fourier amplitudes (see *Methods* [Sec sec2]). In general, the efficiency of the phase determination procedure is sensitive to the apod­ization scheme. In particular, if the effective resolution of the image is set either too low or too high at the outset, the frequency with which solutions are located reduces. The algorithm is initiated with a random phase set, and the consensus envelope from the envelope determination step. The envelope is held fixed for only the first 10 iterations, after which it is updated at each iteration based on the current solution estimate (see *Methods* [Sec sec2]). The initiation of high-resolution phase determination with an approximately correct envelope biases the starting phase set at low resolution in a way that makes the location of the solution much more likely.

At the conclusion of the main procedure (7200 iterations of the *DM* algorithm), the *DM* algorithm and the conventional *ER* algorithm are alternated for an additional 900 iterations, with no apodization applied to the data, as described in *Methods* [Sec sec2], and the run concludes. The first 7200 iterations serve to search the parameter space and bring the solution estimate into the vicinity of the true solution, while the last 900 iterations give convergence to the final solution.

**Figure 5:**
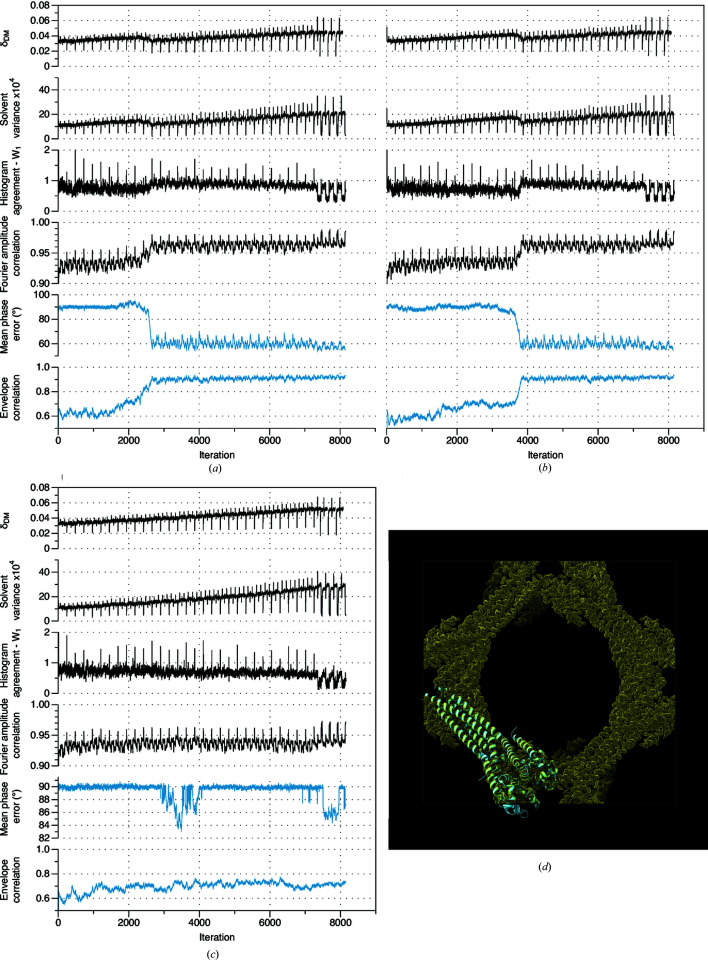
Phase determination for test case 5hk7 (Arrigoni *et al.*, 2016 ▸) (solvent fraction 0.833, 2.95 Å resolution). (*a*) and (*b*) The trajectories of two successful runs. (*c*) The trajectory of an unsuccessful run. These runs were initiated with the same molecular envelope [Fig. 4 ▸(*b*)] but with different random phase sets. Across the first 7200 iterations, the discontinuities apparent every 240 iterations are associated with the steady reduction in data apodization, while the discontinuities apparent every 60 iterations are associated with switching of the *DM* algorithm parameter β between two values. In parts (*a*)–(*c*), plotted from top to bottom, as a function of iteration, are the convergence indicator of the *DM* algorithm (δ_DM_); the variance in the solvent region; the Wasserstein distance between reconstructed and reference histograms in the protein region; the correlation between reconstructed and measured Fourier amplitudes; the weighted mean absolute difference between reconstructed and model phases; and the correlation between the reconstructed and model envelope. The metrics that could be followed during determination of an unknown structure are shown in black, while the metrics that assess agreement with the known solution are shown in blue. (*d*) Consensus electron-density map determined from 10 independent runs, with the molecular structure of the bacterial sodium channel (5hk7) shown in ribbon representation.

Behavior typically observed during the phase determination step is illustrated in Fig. 5 ▸. This shows the trajectories of two successful [Figs. 5 ▸(*a*) and 5 ▸(*b*)] and one unsuccessful [Fig. 5 ▸(*c*)] phasing runs for test case 5hk7 (Arrigoni *et al.*, 2016 ▸) . In the two successful runs, the solution is located near iteration 2200 and 3500 respectively, as indicated by the steady reduction in the mean phase error over the next 300 iterations. Unlike the envelope determination step, location of the solution is indicated by relatively clear perturbations in the agreement metrics for the real and Fourier space constraints, and in the convergence measure for the *DM* algorithm itself. Characteristically, the location of the solution is preceded by a steady increase in the fidelity of the molecular envelope. In the unsuccessful run [Fig. 5 ▸(*c*)], the algorithm begins to advance toward a solution at iteration 3330, and then again at iteration 7500, but in each case regresses, and the solution is not ultimately located. This instability is an inevitable consequence of the good global convergence properties of the algorithm.

While arrival at the true solution is generally indicated by the usual agreement metrics, there are occasional counter examples. For test case 2ja1 (Kosinska *et al.*, 2007 ▸) the solution is rapidly located (often within the first 1000–2000 iterations) and with relatively high frequency (14/20 phase determination runs locating the solution). However, the progression to the solution is not clearly indicated by the conventional agreement metrics, or by the algorithm convergence measure (Supplementary Fig. S2). Hence for determining unknown structures, a clustering procedure is also useful to robustly identify true solutions to the phase retrieval problem.

Clustering of phase sets to determine if the solution has been located is straightforward, with subsequent averaging of the cluster members producing a consensus phase set. As a specific example, we again consider test case 5hk7 [for which three run trajectories are shown in Figs. 5(*a*)–5(*c*) ▸]. In this case, clustering of the phase sets with *DB-SCAN* (threshold mean absolute phase difference ɛ = 40°, *MinPoints* = 2) produces a single cluster of size 10, from a total of 20 runs. As imposed by the distance threshold, the phase sets within this cluster are all very similar (sample circular variance = 0.20, Table 1 ▸). The 10 members of the cluster correspond to the true solution, and have mean differences from the model phases of 54–56° [see *e.g.* Figs. 5(*a*) and 5(*b*) ▸]. The remaining 10 runs did not locate the true solution, and their mean differences with model phases are all close to 90° [see *e.g.* Fig. 5 ▸(*c*)]. Although the space group (*I*222) is achiral, only the solution with the correct hand is generated in this instance. Averaging across the cluster members produces a consensus phase set with mean difference with model phases of 52°, indicating that small residual random errors in the solution estimate are removed by averaging. Fig. 5(*d*) ▸ shows the reconstructed density, together with the refined atomic model from the PDB.

The solution was located for 22 of the 42 test cases, with final mean differences between the consensus and model phases ranging from 34 to 53° (Table 1 ▸). A maximum of one cluster was generated in the chiral space groups (corresponding to the true density), and a maximum of two clusters generated in the achiral space groups (corresponding to the true density, and the inversion of the true density). No false positives were generated (*i.e.* no clusters corresponding to an incorrect solution were identified). *Hence if the phase determination procedure generates two or more highly consistent phase sets, having mean absolute phase difference < 40–50°, this unambiguously indicates that the phase retrieval problem has been solved*. This provides a simple and reliable diagnostic of success, useful in the determination of unknown structures. While conventional metrics, like the correlation between measured and reconstructed Fourier amplitudes, do sometimes indicate that a solution the phase problem has been located (Fig. 5 ▸), the consistency of the final phase sets produced by the algorithm is a much more robust indicator of success.

**Figure 6:**
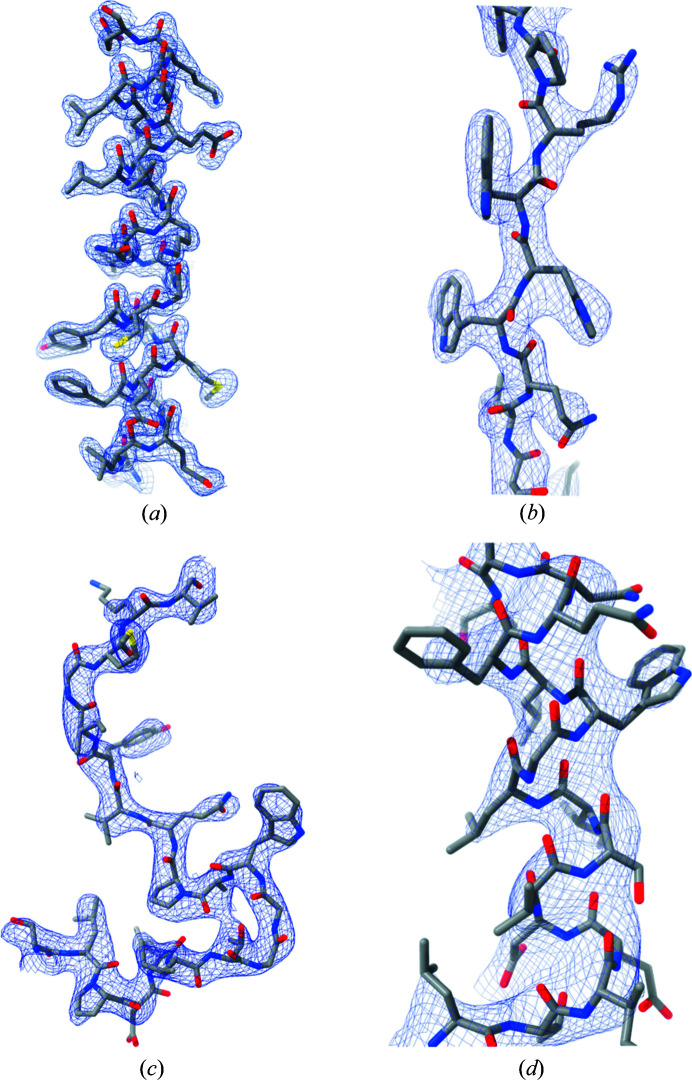
Details of the reconstructed electron density for selected test cases at varying resolution. (*a*) 3u6u (Sundaresan *et al.*, 2012 ▸) (solvent fraction 0.74, 1.92 Å resolution). (*b*) 4bsj (Leppänen *et al.*, 2013 ▸) (solvent fraction 0.74, 2.50 Å resolution). (*c*) 3als (Hatakeyama *et al.*, 2011 ▸) (solvent fraction 0.79, 3.00 Å resolution). (*d*) 4asn (Aylett & Löwe, 2012 ▸) (solvent fraction 0.76, 3.50 Å resolution). In each instance a portion of the reconstructed density is displayed together with the refined atonic model in stick representation. Zoning was applied to visualize the relevant subregion of each map.

In the achiral space groups, if the low-resolution molecular envelope is well discriminated from its inverse (*e.g.* 5hk7, 4fzn, 4asn, 4tpl, Supplementary Table S2) then the hand of the reconstruction is fixed by the initial imposition of the envelope. In contrast, if the low–resolution molecular envelope is similar to its inverse (*e.g.* 4c94, 2xol, 2vvx, 3me2, Supplementary Table S2), densities with either hand emerge. Solutions obtained at varying resolution are illustrated in Fig. 6 ▸. In contrast to the envelope determination step, the success of the phase determination step is clearly tied to the solvent content. Across the 42 test cases, the breakdown is given in Table 2 ▸ [see also Fig. 3 ▸(*b*)].

If the phase determination step is initiated without initial imposition of a low-resolution molecular envelope (*i.e.* a single-stage phase determination procedure is adopted) the result is always a net reduction in efficiency (*i.e.* the solution is located with lower frequency). We executed a single-stage phase determination procedure for three test cases presenting varying levels of overall difficulty. The comparative results are shown in Table 3 ▸. While in some cases, the loss in efficiency will be tolerable, in other cases the loss in efficiency is likely to be severe and could preclude location of the solution in a reasonable amount of time.

Finally, we note that for some test cases where our procedure is presently unable to determine a solution, there are indications that the problem is tractable. One example is 4zmx (Kudo *et al.*, 2016 ▸) (solvent fraction 0.71), where a correct low-resolution phase set repeatedly starts to form during the phase determination step, but the solution is lost as the resolution of the image reconstruction increases throughout the runs. Across the 20 runs, the smallest final mean difference with model phases is 81°. It is likely that further improvements in the parameterization of the procedure will bring such cases within scope of the method.

### 3.4. Practical issues: space group ambiguity and errors in the estimation of the solvent fraction

In real cases there are several additional issues to be dealt with.

One of these is space group ambiguity. There are 11 enantiomorphic space group pairs (Nespolo *et al.*, 2018 ▸) that cannot be discriminated based on the diffraction data alone. In such a case it is necessary to run the calculations in just one of the possible space groups. If the wrong space group is selected, a solution may be located, but the density map will have the wrong hand. For example, if envelope and phasing calculations for test case 3als (Hatakeyama *et al.*, 2011 ▸) (space group *P*6_5_) are carried out in the enantiomorphic space group *P*6_1_ an envelope coalesces which is the mirror image of the true envelope. At the phase determination step a consistent solution is found, however inspection of the density map shows that the helices are left-handed, indicating that the true space group is *P*6_5_.

Potentially more problematic is the issue of the unknown solvent content. In some cases, particularly with small unit cells, there may be no ambiguity in this parameter, as any reasonable assumptions about the crystal packing density will lead to a unique answer (Weichenberger *et al.*, 2015 ▸). However, in general, there may be some uncertainty about the exact number of molecules in the asymmetric unit, and hence the total solvent fraction. We have performed some preliminary exploration of this issue with test case 3als (Hatakeyama *et al.*, 2011 ▸) (space group *P*6_5_, with four molecules in the asymmetric unit, and solvent fraction 0.791). When using the correct solvent fraction, the solution was easy to locate for 3als, with all of the phase determination runs converging to the correct result (Table 1 ▸). The envelope and phasing calculations were repeated using solvent fractions based on the incorrect assumption of three molecules in the asymmetric unit (solvent fraction 0.84) or five molecules in the asymmetric unit (solvent fraction 0.74), both of which are physically plausible.

With the solvent fraction set too low, phase determination was essentially unaffected with 10/10 runs converging to the solution, and a mean absolute difference between consensus and model phases of 39°. This is in fact better than the 44° achieved using the correct solvent fraction (Table 1 ▸). Underestimation of the solvent fraction is consistent with the solution, and hence the result is unsurprising, although the lower solvent fraction does provide a weaker constraint on the density.

With the solvent fraction set too high, phase determination was severely impacted, with only 1/10 runs progressing toward the solution (and that run stalling with a mean absolute difference of 67° with model phases). Since overestimation of the solvent fraction is inconsistent with the solution (it must suppress density in the protein region), this is also expected. The result is diagnostic of overestimation of the solvent fraction.

## 4. Discussion

Solvent flatness has long been known to provide a powerful phase constraint in protein crystallography. We have shown here that the solvent flatness constraint, coupled with information on the protein density value distribution, is strong enough to effectively solve the phase problem *ab initio*, for the majority of high-solvent-content crystals, providing that an algorithm with good global search capabilities is employed. The feasibility of directly phasing diffraction data from high-solvent-content protein crystals has been predicted theor­etically (Millane & Arnal, 2015 ▸) and demonstrated practically (He & Su, 2015 ▸; Jiang *et al.*, 2018 ▸; He & Su, 2018 ▸; Jiang *et al.*, 2019 ▸), but so far no general method has emerged. This study establishes the practicality of direct phasing for high-solvent-content crystals, subject to a reasonable estimate for the solvent fraction. The procedure we have implemented is unsupervised and does not require tuning or case–based decision making. Tested on a randomly selected set of 42 structures at modest resolution, it routinely succeeds when the solvent fraction is greater than 0.70, and is extremely likely to succeed when the solvent fraction is greater than 0.75. Preliminary tests suggest that the procedure is robust to errors in the assumed solvent content of the crystal.

While already providing a viable method for structure determination, it is probable that the approach can be further improved. The results reported here provide a benchmark against which future developments can be evaluated. In particular, the success of the algorithm, at both the envelope determination and phase determination steps, is quite sensitive to the apodization scheme; the parameter β which controls the behavior of the *Difference Map* algorithm; and the filter radius used during molecular envelope determination, amongst other variables. Developing and testing more sophisticated schemes for controlling these parameters is one focus of our current work.

Exactly how far the reach of this algorithm can be extended, without introducing stronger constraints on the density function is presently unclear. About 4.5% of protein crystals are expected to have solvent fraction greater than 0.7, but 19% will have solvent fraction greater than 0.6, and 52% will have solvent fraction greater than 0.5 (Weichenberger & Rupp, 2014 ▸). Hence, even a small lowering of the threshold solvent content for applicability of the method would bring many more structures into reach. There are more powerful image constraints that might be applied in the protein region, the most obvious of these being non-crystallographic symmetry (NCS). NCS is commonplace (Kleywegt, 1996 ▸) and is frequently exploited for conventional phase refinement (Kleywegt & Read, 1997 ▸). This can be seen in Supplementary Table S1, where 57% (24/42) of the test cases have NCS, which was not used as a constraint in our phase determination protocol. However, it is challenging to implement a symmetry constraint in any general fashion, because the nature of any symmetry present, and the position and orientation of any symmetry elements, are all *a priori* unknown. While it might be possible to deduce the order of the rotational symmetry prior to phasing (Blow, 1976 ▸; Sawaya, 2007 ▸), translational parameters, in particular, would need to be co-determined with a symmetry-constrained density, significantly increasing the complexity and difficulty of the problem. There has been some work in this area (He *et al.*, 2019 ▸), but the resulting algorithm was very complicated, and appears unlikely to be generally applicable. If better methods for detecting and exploiting molecular symmetry can be devised, that would greatly increase the reach of the algorithm. However, there may be other constraints, reflecting generic properties of the protein density, that will also prove effective in this regard.

Because of dramatic recent advances in protein structure prediction (Jumper *et al.*, 2021 ▸; Lupas *et al.*, 2021 ▸), molecular replacement is becoming the predominant method for obtaining initial phase estimates in protein crystallography. However, particularly at modest resolution (<2.5 Å) there is the potential for significant model bias (Adams *et al.*, 1999 ▸; DiMaio *et al.*, 2011 ▸) when using this approach. We have shown that *ab initio* phase determination using iterative projection algorithms can be effective with this kind of data, and will produce solutions free of any model bias. It may be that a hybrid procedure, where the algorithm we described is initiated with model-based phase estimates, subject to some limited randomization, would also be effective in reducing or eliminating bias.

Some computational aspects of our approach deserve comment. The procedure employed for *ab initio* phase determination is obviously decomposable into many identical and fully separable subtasks. These are the individual runs, initiated with random phase sets, that are performed during both the envelope and phase determination steps. Parallel­ization of these steps makes the procedure relatively rapid to perform. The implementation of the approach we have publicly released is fully parallelized.

A two–stage procedure has been adopted for reasons of computational efficiency (*i.e.* because it decreases the overall number of iterations of the algorithm required to locate the solution). For some test cases the phase determination problem is relatively easy, and a separate envelope determination step is not strictly necessary, as a randomly initiated phase-determination step, with gradual resolution extension, would converge to the solution often enough to be viable (Table 3 ▸). However, in more difficult cases a single-stage algorithm may converge to the solution extremely sporadically from a completely random starting point. This results in the execution of many lengthy phase-determination runs, the vast majority of which are unsuccessful. However, if an approximate molecular envelope is first determined, and imposed at the very start of the phase determination procedure, efficiency is always improved (Table 3 ▸). A key observation is that a correct envelope often coalesces at low resolution before a full solution to the phase problem is located (Fig. 1 ▸). Hence, the envelope can be efficiently computed at low resolution, and used to effectively bias the initialization of the phase determination procedure at a higher resolution. The envelope determination calculations can be carried out quite rapidly, because a coarse grid can be used to represent the density, and an envelope typically coalesces in a relatively small number of iterations (Fig. 1 ▸).

In summary, we present a general-purpose, unsupervised, *a priori* phasing procedure for diffraction data of modest resolution, applicable to protein crystals with a solvent content greater than about 70%. The method is based on the use of an iterative projection algorithm to solve the global constraint satisfaction problem, with rigorous incorporation of solvent flatness and histogram constraints. Computational efficiency is improved by breaking the problem down into separable envelope-determination and phase-determination stages. Clustering procedures are used to identify and promote correct solutions. At the phase determination step, the emergence of highly consistent phase sets (mean absolute phase difference < 40–50°) from different randomly initiated runs, is a simple and reliable indicator of success. Such phase sets always correspond to the solution. Tests on 42 previously determined structures (solvent fraction 0.60–0.85, resolution 1.9–3.5 Å), selected at random, demonstrate the effectiveness of the algorithm. The implementation is based on the Clipper crystallographic library (Cowtan, 2003 ▸) and the code is publicly available. The algorithm has immediate practical application to crystals with high solvent content. With the incorporation of additional constraints on the protein density, the approach has potential to be effective for crystals with much lower solvent content.

## Supplementary Material

Supplementary figures and tables. DOI: 10.1107/S2052252522006996/jt5063sup1.pdf

## Figures and Tables

**Table 1:** Results of phase set clustering for the 22 successful test cases A dash means that no result was generated. N/A means a result cannot be generated because the symmetry does not allow it.

			True solution			Inverse of true solution			
PDB ID/solvent fraction/resolution (Å)	Space group	Total no. of phase determination runs	Cluster size	Sample circular variance	Mean absolute difference between consensus and model phases (°)	Cluster size	Sample circular variance	Mean absolute difference between consensus and inverted model phases (°)	Successful runs†
6p72/0.84/3.28	*P*6_5_	20	20	0.25	44	N/A	N/A	N/A	100
5hk7/0.83/2.95	*I*222	20	10	0.20	52	–	–	–	50
4c94/0.83/3.00	*C*222_1_	20	5	0.13	41	13	0.15	39	90
2w88/0.81/2.89	*P*3_1_12	20	19	0.18	47	N/A	N/A	N/A	95
2x0l/0.81/3.00	*I*222	20	3	0.09	41	16	0.12	40	95
3lii/0.80/3.20	*P*6_1_	20	19	0.18	40	N/A	N/A	N/A	95
4fzn/0.80/2.86	*P*6_3_22	20	9	0.14	49	–	–	–	45
2rha/0.80/2.10	*P*4_3_2_1_2	20	14	0.15	41	N/A	N/A	N/A	70
3als/0.79/3.00	*P*6_5_	20	20	0.23	44	N/A	N/A	N/A	100
2w4m/0.77/2.60	*P*3_2_21	20	17	0.18	46	N/A	N/A	N/A	85
2ja1/0.77/2.80	*I*4_1_22	20	14	0.26	49	–	–	–	70
4asn/0.76/3.50	*H*32	20	–	–	–	11	0.13	48	55
4pqe/0.75/2.90	*P*3_1_12	20	13	0.18	40	N/A	N/A	N/A	65
4c5h/0.75/3.20	*P*3_1_21	20	15	0.15	42	N/A	N/A	N/A	75
2vvx/0.75/2.75	*H*3	20	14	0.16	43	6	0.15	44	100
3u6u/0.74/1.92	*P*6_5_	20	12	0.13	34	N/A	N/A	N/A	60
4bsj/0.74/2.50	*P*3_1_21	20	5	0.20	53	N/A	N/A	N/A	25
3me2/0.74/2.80	*P*6_3_	20	10	0.13	47	10	0.13	40	100
4bex/0.73/2.80	*P*3_2_21	20	16	0.20	47	N/A	N/A	N/A	80
5b2c/0.73/2.24	*P*6_1_	20	17	0.20	42	N/A	N/A	N/A	85
4tpl/0.73/2.90	*P*321	20	–	–	–	4	0.19	52	20
3mf0/0.71/3.10	*P*3_1_21	20	5	0.17	48	N/A	N/A	N/A	25

† Expressed as a % of the total number of runs.

**Table 2:** Overall success rate for the phase determination procedure as a function of solvent content

Solvent fraction	Raw number of structures determined	Success rate (%)
0.75 < solvent fraction < 0.85	13/15	87
0.70 < solvent fraction < 0.75	9/15	60
0.60 < solvent fraction < 0.70	0/12	0

**Table 3:** The effect of initial imposition of the molecular envelope of the efficiency of the phase determination step

	Frequency of convergence to the correct solution (allowing for inversion)	
PDB ID/solvent fraction/resolution (Å)	With imposition of the estimated envelope (two-stage procedure)	Without imposition of the estimated envelope (single-stage procedure)
4bex/0.73/2.80	16/20	4/20
4bsj/0.74/2.50	5/20	1/20
4tpl/0.73/2.90	4/20	0/20
