# Supplementary material for: A general method for directly phasing diffraction data from high-solvent-content protein crystals
Source: IUCrJ. 2022 Aug 13;9(Pt 5):648–65. doi: 10.1107/S2052252522006996 (PMC9438493; doi:10.1107/S2052252522006996)
Supplement: Supplementary file 1 [file m-09-00648-sup1.pdf]

IUCrJ

Volume 9 (2022)

Supporting information for article:

A general method for directly phasing diffraction data from high-solvent-content protein crystals

Richard Lawrence Kingston and Rick P. Millane

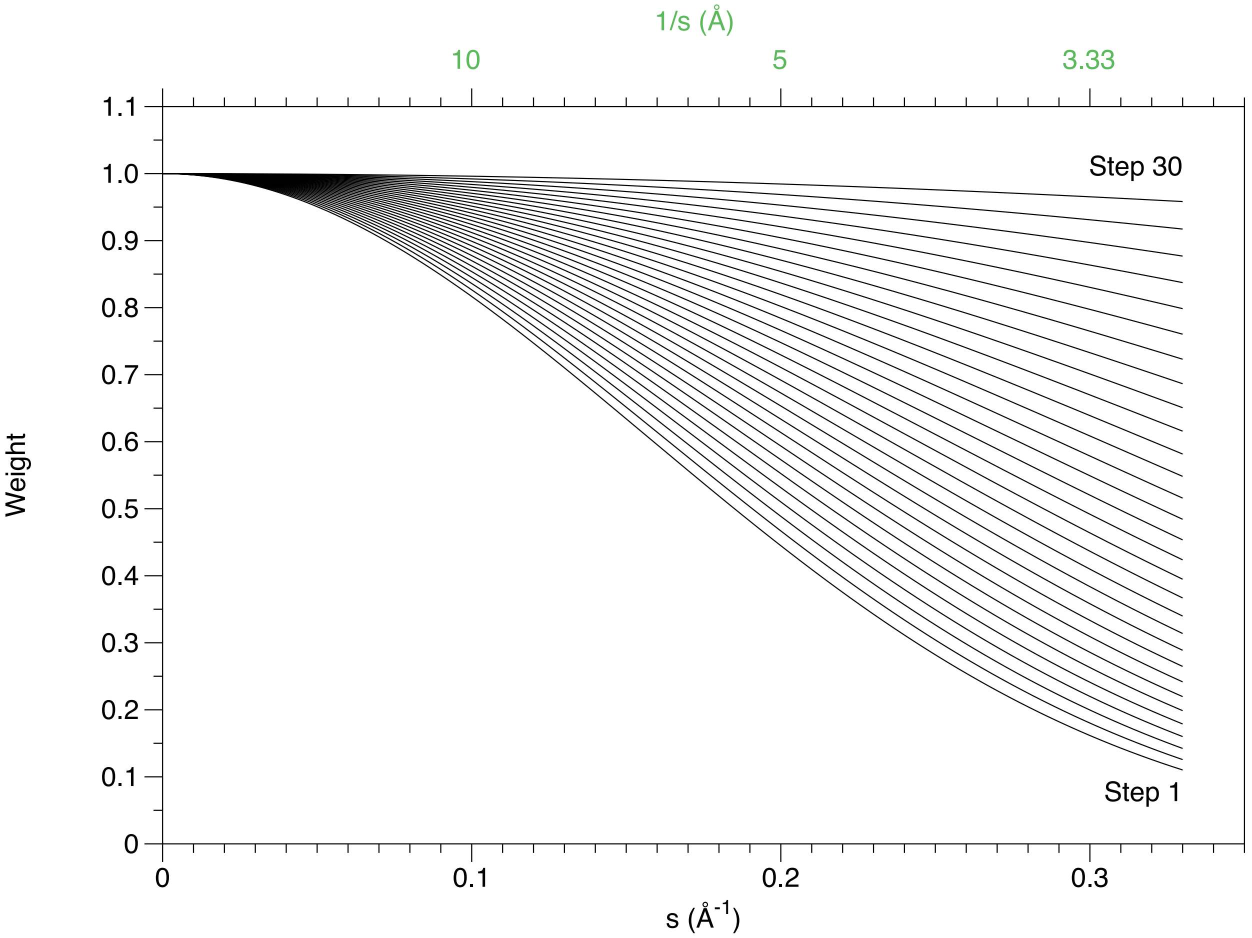

Fig. S1 The Gaussian weighting functions (Eqn 4) used for data apodization during phase determination, as applied to a 3 Å resolution data set. At step 1 the Gaussian function has σ = 0.157 Å^{-1} (hence reaching 1/100th of maximum height at 2.1 Å resolution). At step 30 the Gaussian function has σ = 1.13 Å^{-1} (hence reaching 1/100th of maximum height at 0.29 Å resolution). At each apodization step the definite integral of the Gaussian function (evaluated between s=0 and s= 0.33 Å^{-1}) increases by a constant amount.

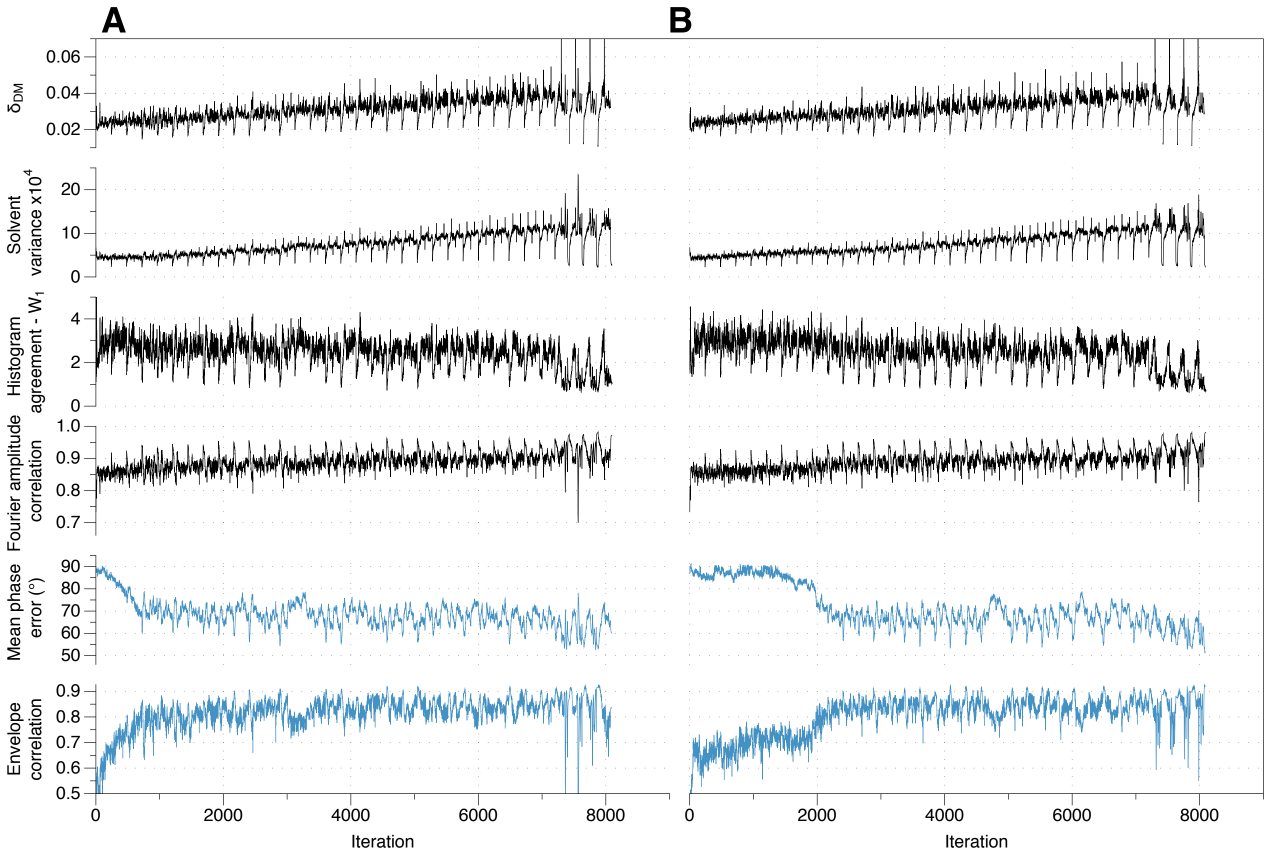

Figure S2. Phase determination for test case 2JA1 (solvent fraction 0.77, 2.80 Å resolution). (A) and (B) The trajectories of two successful runs. Convergence to the solution, which begins immediately in run (A) and around iteration 1800 in run (B) is not indicated by significant changes in the real or Fourier space agreement metrics. Plotted from top to bottom, as a function of iteration, are the convergence indicator of the DM algorithm (δ_{DM}); the variance in the solvent region; the Wasserstein distance between reconstructed and reference histograms in the protein region; the correlation between reconstructed and measured Fourier amplitudes; the weighted mean absolute difference between reconstructed and model phases; and the correlation between the reconstructed and model envelope. The metrics that could be followed during determination of an unknown structure are shown in black, while the metrics that assess agreement with the known solution are shown in blue.

Table S1: Detail of the 42 test cases used in the study

PDB ID	Solvent Fraction	Resolution (Å)	Space Group	Overall Isotropic B: Estimated from data (Å²)	Mean Isotropic B: Evaluated from model (Å²)	Asymmetric unit contents	Reference structure for histogram matching [Resolution (Å), Estimated B-factor (Å²)]
4AVT	0.848	3.20	$P4_3 2_1 2$	74.60	NR	10x 204 aa chains	4D4K [3.24, 68.95]
6P72	0.836	3.28	$P6_5$	95.33	62.17	2x 429 aa chains	4C0P [2.95, 84.61]
5HK7	0.833	2.95	I222	93.19	91.41	4x 152aa chains	4C0P [2.95, 84.61]
4C94	0.826	3.00	$C222_1$	60.19	74.66	5x 161aa chain	5WOR [2.77, 63.56]
2W88	0.810	2.89	$P3_112$	47.04	29.10	3x 106 aa chains	5G1U [2.57, 44.96]
2X0L	0.809	3.00	I222	70.85	64.13	1x 734 aa,1x 133aa,1x16 aa chain	4D4K [3.24, 68.95]
3LII	0.803	3.20	$P6_1$	74.66	76.54	2x 540 aa chains	4D4K [3.24, 68.95]
4FZN	0.799	2.86	$P6_322$	94.57	98.70	1x 283 aa chain	4C0P [2.95, 84.61]
2RHA	0.798	2.10	$P4_32_12$	39.61	35.68	1x 213 aa chain	5FOY [2.35, 39.94]
3ALS	0.791	3.00	$P6_5$	48.43	39.87	4x 157 aa chains	1XF9 [2.70, 49.27]
2W4M	0.770	2.60	$P3_221$	49.32	23.26	1x 270 aa chain	1XF9 [2.70, 49.27]
2JA1	0.770	2.80	$I4_122$	75.59	57.80	1x 197aa chain	4C0P [2.95, 84.61]
1DOV	0.770	3.00	H32	65.15	69.50	1x 181 aa chain	4GEX [2.80, 66.34]
4ASN	0.760	3.50	H32	157.80	144.50	3x 101 aa chains	3ZXU [3.70, 190.0]
4PQE	0.754	2.90	$P3_112$	44.63	30.23	1x 543 aa chain	5G1U [2.57, 44.96]
4C5H	0.748	3.20	$P3_121$	84.90	46.20	1x 451 aa, 1x 135 aa chains	4C0P [2.95, 84.61]
2VVX	0.747	2.75	H3	61.80	81.77	2x 162 aa chains	5WOR [2.77, 63.56]
3RUJ	0.744	2.10	$I4_1$	40.17	NR	1x 296 aa chain	5FOY [2.35, 39.94]
3U6U	0.743	1.92	$P6_5$	30.30	37.90	2x 269 aa chains	4KYC [1.95, 27.73]
3MIT	0.743	2.32	$P3_221$	41.54	48.20	2x 141 aa chains	5FOY [2.35, 39.94]
4RBN	0.742	3.05	$P6_5$	59.43	NR	4x 794 aa chains	5WOR [2.77, 63.56]
4BSJ	0.741	2.50	$P3_121$	65.07	61.20	1x 232 aa chain	4GEX [2.80, 66.34]
3ME2	0.738	2.80	$P6_3$	50.23	58.14	1x 171 aa, 1x 276 aa chains	1XF9 [2.70, 49.27]
4BEX	0.734	2.80	$P3_221$	70.17	68.75	1x 181 aa chain	4GEX [2.80, 66.34]
5B2C	0.726	2.24	$P6_1$	37.20	NR	2x 489 aa chains	5FOY [2.35, 39.94]
4TPL	0.726	2.90	P321	80.89	100.30	2x 377aa chains	4C0P [2.95, 84.61]
3ME6	0.708	3.10	P3	52.94	42.20	4x 476 aa chains	4D82 [3.20, 55.27]
3MCY	0.708	2.90	$P4_132$	52.43	48.32	4x 181 aa chains	4D82 [3.20, 55.27]
4ZMX	0.706	3.10	$P6_3$	53.82	60.37	2x 213 aa chains	4D82 [3.20, 55.27]
3MF0	0.706	3.10	$P3_121$	99.29	100.54	2x 432 aa chains	4C0P [2.95, 84.61]
2Y4U	0.692	3.20	$P3_12$	115.30	118.54	1x 450 aa chain	4C0P [2.95, 84.61]
4GBG	0.687	2.90	$P6_1$	50.02	50.60	2x 269aa chains	1XF9 [2.70, 49.27]
3M7P	0.684	2.50	$P3_221$	46.40	60.77	1x 308 aa chain	5G1U [2.57, 44.96]
2VUH	0.673	2.50	$P3_212$	48.91	53.47	1x 139 aa chain	1XF9 [2.70, 49.27]
4LXF	0.669	2.50	$P3_212$	66.55	69.24	2x 620 aa chains	4GEX [2.80, 66.34]
4BXE	0.665	2.95	$P4_12_12$	51.93	NR	2x 255aa chains	1XF9 [2.70, 49.27]
4GBE	0.661	2.66	$P3_121$	48.02	46.90	3x 284 aa chains	1XF9 [2.70, 49.27]
4CAZ	0.660	2.55	$P3_121$	41.08	38.50	2x 490 aa chains	5FOY [2.35, 39.94]
4ZLE	0.640	2.10	$P3_121$	30.95	30.55	1x 796 aa chain	4KYC [1.95, 27.73]
4ZMN	0.611	2.60	$P6_522$	43.53	41.77	1x 231 aa chain	5G1U [2.57, 44.96]
4ZQK	0.610	2.45	$P3_121$	47.48	57.57	1x 115 aa, 1x 118 aa chains	1XF9 [2.70, 49.27]
1CIW	0.603	2.70	$P2_1 2_1 2$	50.26	29.50	4x 236 aa chains	1XF9 [2.70, 49.27]

Table S2. Results of Molecular Envelope determination

	All 50 Envelopes		Clusters		Cluster Consensus			
PDB ID / Solvent Fraction / Resolution(Å)	Median cc with model envelope	Maximum cc with model envelope	Size	Mean cc with model envelope	Number of connected sets[1]	Deviation from solvent volume fraction	cc with model envelope	cc with model envelope following inversion
4AVT / 0.85 / 3.20	**0.105**	**0.191**	20	**0.069**	4	0.10	**0.121**	-
6P72 / 0.84 / 3.28[2]	**0.050**	**0.310**	5	**0.255**	3	0.10	**0.341**	-
			24	**0.006**	1	0.11	**-0.107**	-
5HK7 / 0.83 / 2.95	**0.358**	**0.547**	11	**0.460**	2	0.04	**0.603**	**0.447**
			20	**0.356**	2	0.03	**0.419**	**0.568**
4C94 / 0.83 / 3.00	**0.080**	**0.594**	10	**0.435**	8	0.03	**0.584**	**0.625**
			15	**-0.151**	6	0.05	**-0.173**	**-0.174**
2W88 / 0.81 / 2.89	**0.310**	**0.482**	28	**0.348**	3	0.11	**0.497**	-
2X0L / 0.81 / 3.00	**0.418**	**0.552**	26	**0.458**	2	0.04	**0.610**	**0.637**
3LII / 0.80 / 3.20	**0.079**	**0.386**	16	**0.306**	6	0.12	**0.559**	-
4FZN / 0.80 / 2.86	**0.456**	**0.797**	13	**0.696**	1	0.02	**0.799**	**0.596**
			5	**0.528**	1	0.01	**0.577**	**0.779**
2RHA / 0.80 / 2.10	**0.761**	**0.867**	14	**0.832**	1	-0.00	**0.858**	-
3ALS / 0.79 / 3.00	**0.363**	**0.757**	15	**0.621**	1	0.02	**0.786**	-
2W4M / 0.77 / 2.60	**0.473**	**0.817**	23	**0.597**	1	0.01	**0.721**	-
2JA1 / 0.77 / 2.80	**0.216**	**0.464**	22	**0.291**	4	0.12	**0.557**	**0.514**
1DOV / 0.77 / 3.00	**0.181**	**0.367**	21	**0.171**	3	0.08	**0.228**	**0.267**
4ASN / 0.76 / 3.50	**0.383**	**0.830**	10	**0.427**	1	-0.01	**0.432**	**0.861**
			5	**0.230**	1	0.00	**0.243**	**0.364**
4PQE / 0.75 / 2.90	**0.325**	**0.644**	16	**0.494**	1	0.04	**0.715**	-
4C5H / 0.75 / 3.20	**0.117**	**0.781**	11	**0.683**	1	0.01	**0.817**	-
2VVX / 0.75 / 2.75	**0.275**	**0.481**	11	**0.398**	1	0.09	**0.612**	**0.543**
			6	**0.302**	3	0.07	**0.399**	**0.497**
			6	**-0.011**	3	0.11	**-0.093**	**-0.102**
3RUJ / 0.74 / 2.10	**0.094**	**0.275**	6	**0.177**	1	0.11	**0.254**	**-0.042**
			25	**0.068**	112	0.25	**0.002**	**0.063**
3U6U / 0.74 / 1.92	**0.053**	**0.150**	6	**0.085**	1	0.11	**0.114**	-
			5	**0.016**	15	0.13	**0.000**	-
3MIT / 0.74 / 2.32	**0.083**	**0.360**	7	**0.289**	1	0.08	**0.380**	-
			5	**0.239**	1	0.06	**0.332**	-
			17	**-0.089**	1	0.12	**-0.189**	-
4RBN / 0.74 / 3.05[2]	**0.162**	**0.276**	8	**0.222**	1	0.10	**0.386**	-
			17	**-0.050**	1	0.15	**-0.192**	-
4BSJ / 0.74 / 2.50	**0.160**	**0.563**	19	**0.286**	3	0.11	**0.359**	-
			4	**0.171**	3	-0.05	**0.221**	-
			5	**-0.021**	1	0.02	**-0.081**	-
3ME2 / 0.74 / 2.80	**0.570**	**0.771**	10	**0.667**	1	0.02	**0.786**	**0.710**
			13	**0.631**	1	0.01	**0.738**	**0.827**
4BEX / 0.73 / 2.80	**0.344**	**0.745**	17	**0.577**	2	0.04	**0.769**	-
5B2C / 0.73 / 2.24	**0.424**	**0.606**	22	**0.509**	1	0.05	**0.672**	-
4TPL / 0.73 / 2.90	**0.094**	**0.489**	16	**0.240**	1	0.06	**0.369**	**0.477**
			13	**-0.024**	1	0.10	**-0.143**	**-0.129**

	All 50 Envelopes		Clusters		Cluster Consensus			
PDB ID / Solvent Fraction / Resolution(Å)	Median cc with model envelope	Maximum cc with model envelope	Size	Mean cc with model envelope	Number of connected sets[1]	Deviation from solvent volume fraction	cc with model envelope	cc with model envelope following inversion
3ME6 / 0.71 / 3.10	**0.112**	**0.276**	26	**0.090**	1	0.17	**0.085**	**0.068**
3MCY / 0.71 / 2.90	**0.279**	**0.503**	20	**0.386**	1	0.04	**0.587**	-
4ZMX / 0.71 / 3.10	**0.168**	**0.467**	16	**0.350**	3	0.08	**0.576**	**0.596**
			5	**0.182**	1	0.06	**0.225**	**0.225**
			5	**-0.055**	8	0.08	**-0.130**	**-0.127**
3MF0 / 0.71 / 3.10	**-0.036**	**0.391**	9	**0.297**	2	0.10	**0.454**	-
			4	**-0.110**	1	-0.01	**-0.176**	-
			13	**-0.113**	1	0.14	**-0.274**	-
2Y4U / 0.69 / 3.20	**0.270**	**0.480**	24	**0.311**	1	0.07	**0.444**	**0.407**
4GBG / 0.69 / 2.90	**0.227**	**0.602**	17	**0.500**	3	0.02	**0.609**	-
3M7P / 0.68 / 2.50	**0.074**	**0.282**	19	**0.131**	3	0.21	**0.275**	-
			5	**0.102**	2	0.13	**0.088**	-
			5	**0.026**	1	0.12	**-0.001**	-
2VUH / 0.67 / 2.50	**0.370**	**0.579**	17	**0.367**	1	0.01	**0.409**	-
			8	**0.364**	1	-0.01	**0.390**	-
4LXF / 0.67 / 2.50	**0.282**	**0.449**	27	**0.326**	1	0.06	**0.555**	-
4BXE / 0.66 / 2.95	**0.257**	**0.468**	6	**0.372**	1	-0.03	**0.463**	-
			10	**0.339**	1	0.00	**0.439**	-
			10	**0.113**	2	0.02	**0.116**	-
4GBE / 0.66 / 2.66	**0.082**	**0.325**	16	**0.181**	1	0.08	**0.341**	-
			7	**0.022**	1	0.08	**0.001**	-
4CAZ / 0.66 / 2.55	**0.161**	**0.444**	14	**0.344**	1	0.05	**0.566**	-
			12	**0.105**	1	0.05	**0.143**	-
4ZLE / 0.64 / 2.10	**0.076**	**0.313**	15	**0.186**	1	0.13	**0.280**	-
			17	**-0.061**	1	0.16	**-0.253**	-
4ZMN / 0.61 / 2.60	**0.025**	**0.350**	7	**0.120**	1	0.02	**0.100**	-
			18	**-0.134**	1	0.06	**-0.317**	-
4ZQK / 0.61 / 2.45	**0.243**	**0.639**	11	**0.535**	1	0.00	**0.628**	-
			5	**-0.058**	1	0.01	**-0.080**	-
1CIW / 0.60 / 2.70	**0.059**	**0.173**	6	**0.102**	1	-0.04	**0.132**	**0.096**
			15	**0.075**	1	0.17	**0.095**	**0.077**
			6	**0.023**	1	-0.05	**0.001**	**-0.005**

[1] The number of connected sets in the molecular envelope was calculated using the algorithm of Hunt, Vellieux & Deisenhofer (1997) Acta. Cryst. D 53(4):434–7.
[2] Clustering performed with the threshold distance, ε, based on the 6th percentile of the distribution of pairwise distances. If the standard threshold (4th percentile of the distance distribution) was employed the correct cluster was eliminated in this case. Controls show that if the number of input envelopes were increased, the standard threshold could have been employed, generating essentially equivalent results to those displayed.
